# The Structure of the Maxillary Bones

**Published:** 1895-08

**Authors:** M. H. Cryer

**Affiliations:** Philadelphia.


ARTICLE V.

### THE STRUCTURE OF THE MAXILLARY BONES.

BY DR. M. H. CRYER, PHILADELPHIA.

Dr. Cryer showed that the relations of the opening of the maxillary sinus are not what they have been hitherto understood to be; the roots of the great majority of premolar and molar teeth pass up into the antrum, only covered with a slight casing of bone, and in diseases of these teeth, abscesses will form in the antrum, the products of which will pass into the nasal chamber, producing catarrh, and other nasal troubles.

These and other facts, which open out new fields of investigation in dentistry and throw valuable light on the diagnosis of catarrh, were illustrated by means of the stereopticon. The front teeth were shown with the apex of their roots also penetrating the floor of the nasal chamber, producing various unsuspected results and Dr. Cryer demonstrated that in the lower jaw, the so-called inferior dental canal is really a cribiformed tube, suspended in the cancessated portion of the jaw, which constitutes a factor of supreme importance in the treatment of the teeth. The paper produced a sensation, and as one after another of the old landmarks of dentistry were swept away, there was much excitement.

The members of the association, however, were not altogether unprepared for new developments of the subject, for one of the significant features of its convention and that of the New Jersey State Dental Society, which was held in the same hall during the first three days of August, was a sense of expectancy. Papers on the application of electricity to dentistry were more numerous than at any previous convention, and the feeling of helplessness with which certain dental troubles have come to be attacked, even by the most skillful practitioners, is giving way to a hopefulness, or rather a belief, that electricity will form a powerful addition to the means by which the standard of the profession will soon be immeasurably raised. As one of the delegates put it: "We have not got far with electricity, but we feel that there are infinite possibilities in it for the strengthening of our resources. Ii may possibly prove to be the specific we have long been looking for for pyorrhea alveolaris, the mysterious diseased condition of the tooth in which the socket is destroyed and the tooth drops out. There are but few drugs of the classes of escharotics, antiseptics, and astringents which have not been tried as remedies. Each one has had its advocates, subscribers to its efficiency, and just as great a number have declared the same agent worthless. Many of the drugs have been described as almost specific in their action. Undoubtedly, a specific is what is wanted, but the first step to finding a specific remedy is to ascertain that the disease itself is specific. So that, in the same way as the use of certain electric currents has been the means of defining subtle forms of spinal disease, which previously baffled all attempts at diagnosis, we may possibly find in electricity the specific agency for the diagnosis and cure of pyorrhea, which is significant of, it may be, a dozen or more disease conditions."

That the science of dentistry is appreciably widening its scope is shown by the general understanding in the profession that the word "dentistry" has come to be too restricted to express the conditions of dental practice, and that the word "stomatology" will in future be substituted for it. Much emphasis was laid in the discussions which took place at Asbury Park on the deplorable fact that the general public is not educated up to a proper appreciation of dental science, and upon the necessity of agitation in the matter of school-teaching of knowledge relative to the teeth and their preservation. The statistics lately compiled of the state of the teeth of children in public schools throw an important light on the degeneration of the standard of health in young people of late years, and it is admitted that the care of teeth must form an important part of the hygiene of the future.

At a dinner of the dentists an insurance company representative, who was called upon to respond to a toast, said that to such an extent were insurance companies convinced of the importance of the soundness of the teeth in the determination of the fitness of applicants for insurance, that they were now giving teeth inspection the first place in the list of tests. Heretofore the state of the lungs, the heart, and the kidneys had been the primary considerations, but henceforth the examination of the mouth will be the first thing that the inspecting medical officer will proceed to in the case of insurance applicants. What was the use of anxiety as to the power of the driving wheel, the fit of the valve, and the stroke of the piston of the engine if the condition of the furnace were neglected, and insufficient provision were made for the proper combustion of the fuel on which the working of the whole machine depended?

In regard to the too general lack of appreciation, even among educated people, of the importance of early attention to the teeth, a clinician gave an instance which came within his experience just before attending the convention. A patient whom he had known many years told him that he was about to enter his son for the navy. The dentist suggested that the boy's teeth be looked to forthwith. When the parent questioned the necessity, he was informed that no matter what a candidate's general qualifications may be, unless his teeth are up to a standard he cannot enter either the army or the navy. After the examination which followed the dentist, who found the boy's teeth in a lamentable condition, had to tell the parent that, although everything possible would be done in the way of restoring the teeth, the chances of the boy's being accepted were about 3 to 2. He had urged attention being given to the boy's teeth for the last five years, but the boy simply refused to allow it, saying "he couldn't be bothered," and the parents had failed to do their duty and insist that the examination be made.

On a line with this experience was the paper of Dr. Richard C. Newton of Montclair, N. J., on "What shall be done for the teeth of the poor?" Dr. Newton showed that the question of how the poor can be induced to save their teeth is one of wide and vital importance. He had found that the teeth of the very poor are almost universally bad, and in most cases practically the only attention the teeth ever get is when they are pulled out. Bad and insufficient diet lowers the vital forcer, dental caries sets in, and with it a tendency to eschew proper foods and seek for softer and more highly seasoned dishes. This extends the mischief, for working people should eat food that requires considerable mastication—strong food, so called—like the black bread and beans upon which German peasants thrive so well. Poor people will not go to a dentist, even when urged to do so by their medical adviser, because of the expense. Dr. Newton believes that in a comparatively few years, such facilities will be provided that the poor will be able to have their teeth looked to gratuitously. He proposed that the New-Jersey State Dental Society should set the ball rolling, and celebrate this, the twenty-fifth year of its existence, by inaugurating a movement for the establishment of free dental clinics for the poor, and strengthen the bond of union that should exist between all the branches of the profession.—*New York Times.*

